# Regulation of human telomerase splicing by RNA:RNA pairing

**DOI:** 10.1038/ncomms4306

**Published:** 2014-02-28

**Authors:** Mandy S. Wong, Jerry W. Shay, Woodring E. Wright

**Affiliations:** 1 UT Southwestern Medical Center, Department of Cell Biology, 5323 Harry Hines Boulevard, Dallas, Texas 75390-9039, USA

## Abstract

Telomerase adds telomeric repeats onto chromosome ends and is almost universally upregulated in human cancers. Here we demonstrate that RNA:RNA pairing regulates splicing of the catalytic subunit of human telomerase (TERT). Human alleles contain a variable number of 38 bp repeats within *TERT* intron 6 (>1 kb from exon–intron junctions). At least nine repeats are required for generating the major non-functional ‘minus beta’ isoform, which skips exons 7 and 8. RNA:RNA pairing between the repeats and the pre-mRNA might bring exons 6 and 9 closer, thereby promoting exon skipping. To demonstrate this, we show that mutations within the repeat that abolish exon skipping are corrected by compensatory mutations in the pre-mRNA. This study thus identifies RNA:RNA pairing by repetitive sequences as a novel form of alternative splicing regulation in a gene crucial for cancer survival and sheds new light on functional roles for short repetitive sequences embedded deep within introns throughout the genome.

## Abstract (web summary)

Telomerase activity can be regulated by alternative splicing of its catalytic subunit TERT. Here, Wong *et al.* demonstrate that *TERT* splicing is regulated via RNA:RNA pairing of repetitive intronic sequences with the pre-mRNA, thus revealing a new function for conserved elements embedded within introns.

## Graphical abstract

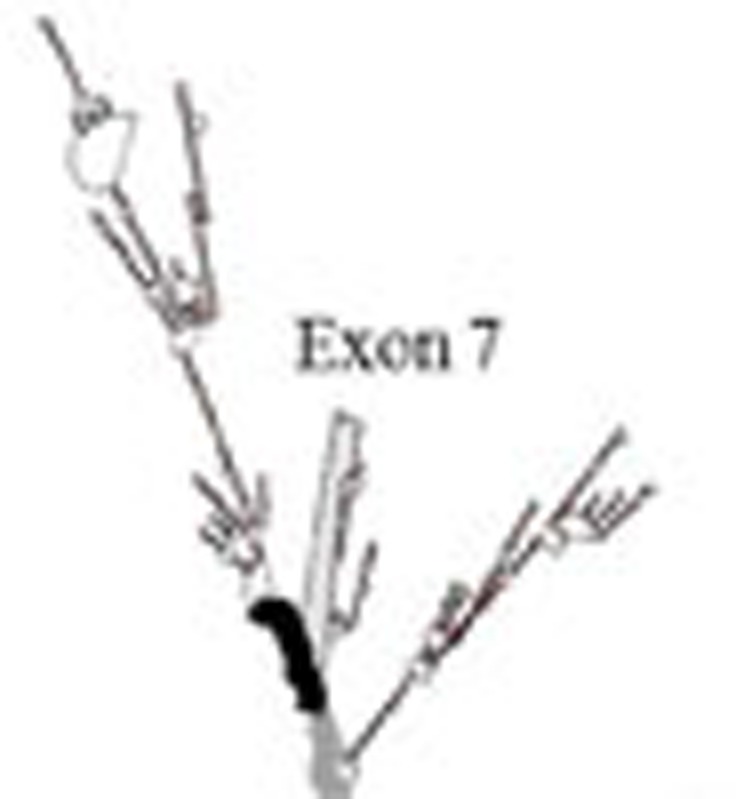

Alternative splicing of pre-mRNA allows for the generation of vast protein diversity from a limited amount of genetic information^1^. Splice choice decisions encompass a large variety of mechanisms and are intricately related to other aspects of transcription^2^. Deregulation of splicing factor expression has been linked to a variety of human disorders including cancer^3^. Human cells have telomeres containing TTAGGG repeats at the ends of chromosomes that prevent the ends from being recognized as DNA damage^4^. In normal cells, telomeres progressively shorten with each cell division ultimately leading to replicative senescence^5^,^6^,^7^. Cancer cells upregulate telomerase activity to bypass or overcome these limits. Introduction of the catalytic subunit of telomerase (TERT) into normal cells prevents replicative senescence, an initial barrier to the accumulation of mutations as part of cancer initiation and progression^8^,^9^. The mechanism(s) regulating telomerase activity are poorly understood but are in part regulated by alternative splicing of the catalytic subunit TERT^10^,^11^. Major efforts to develop telomerase inhibitors as a cancer therapeutic have been largely unsuccessful. Manipulating telomerase splicing to produce non-functional products would provide an alternative approach for inhibiting telomerase activity.

RNA:RNA pairing is a splicing mechanism used for mutually exclusive exon choice in a few insects (such as *Dscam* in *Drosophila melanogaster* and related species)^12^, and until very recently it had not been seen in mammals^13^, where it has been recently reported to control exon choice for a single gene. Human splicing factor (*SF1*, zinc finger protein 162:ZFM162) is the only other mammalian gene for which RNA:RNA pairing has been demonstrated. Previously, we have identified a region within intron 6 of *hTERT* pre-mRNA that contains a variable number of 38 bp repeats, that lies >1 kb from exon–intron junctions, and is only conserved in old but not new world primates or other mammals. This block of repeats in intron 6 is necessary to promote exons 7 and 8 skipping, therefore producing the major non-functional ‘minus beta’ TERT isoform. Although once believed to be ‘junk’ DNA, variable number of tandem repeats (VNTRs) dispersed throughout the genome have been found to have several diverse biological functions^14^.

In this study, we show that a block of repeats promotes exon skipping through RNA:RNA pairing between the repeat sequences and the distal portion of the *TERT* pre-mRNA. We first mutated the repeat sequence to abolish exon skipping and then restored exon skipping by creating compensatory mutations in the pre-mRNA. RNA:RNA pairing through a repetitive sequence deep within an intron that shows conservation in only a small mammalian clade represents completely novel aspects of the RNA:RNA pairing mechanism. This study therefore establishes a novel form of alternative splicing regulation by RNA:RNA pairing through short repetitive sequences and provides additional insights on functions for conserved elements embedded deep within introns.

## Results

### An intronic repetitive element promotes *TERT* exon skipping

**Figure 1:**
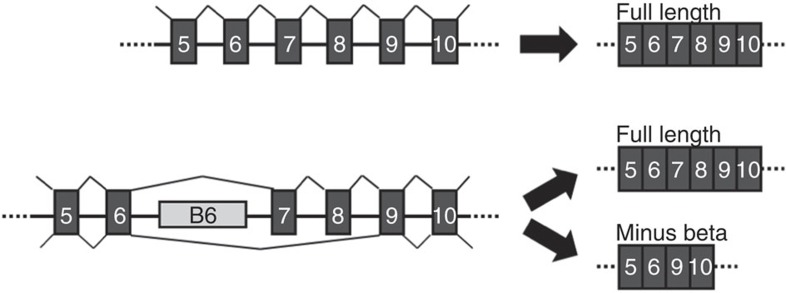
A distal block of repeats in intron 6 is necessary for *TERT* alternative splicing. (**a**) Splicing of *TERT* pre-mRNA (not to scale) without the block of repeats in intron 6 results in exclusively full-length splicing, where all exons are included in the final transcript. (**b**) Splicing of *TERT* pre-mRNA in the presence of the block of repeats in intron 6 produces both the full-length transcript and an alternatively splice transcript called minus beta as a result of the skipping of exons 7 and 8. The minus beta splice form is non-functional because exons 7 and 8 are in the reverse transcriptase domain and required for telomerase activity.

**Figure 2:**
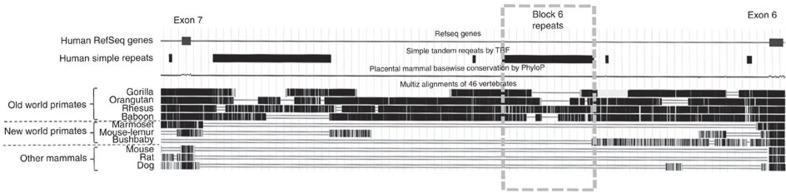
Block 6 repeats are only present in old-world primates. *TERT* sequences from different species were aligned against the human sequence using the UCSC genome browser^20^. The human Block 6 repeats are outlined using a grey dashed box. Variable numbers of these repeats are present in different old-world primates, but they are not present in other mammals.

Telomerase pre-mRNA transcripts are alternatively spliced into many isoforms, but only the full-length transcript containing all 16 exons is capable of encoding the functional enzyme^10^. The skipping of exons 7 and 8 produces the major non-functional isoform called minus beta. Using a minigene stably expressed in a human cancer cell line, we previously identified an ∼1 kb region of 38 bp repetitive elements within intron 6 that is highly conserved in old world but not in new world primates and is required for exons 7 and 8 skipping to make the minus beta isoform^15^ (Figs 1 and 2). Interestingly, this regulatory sequence lies unusually far away (>1 kb) from flanking exon–intron junctions.

### The repeats are predicted to pair with *TERT* pre-mRNA

**Figure 3:**
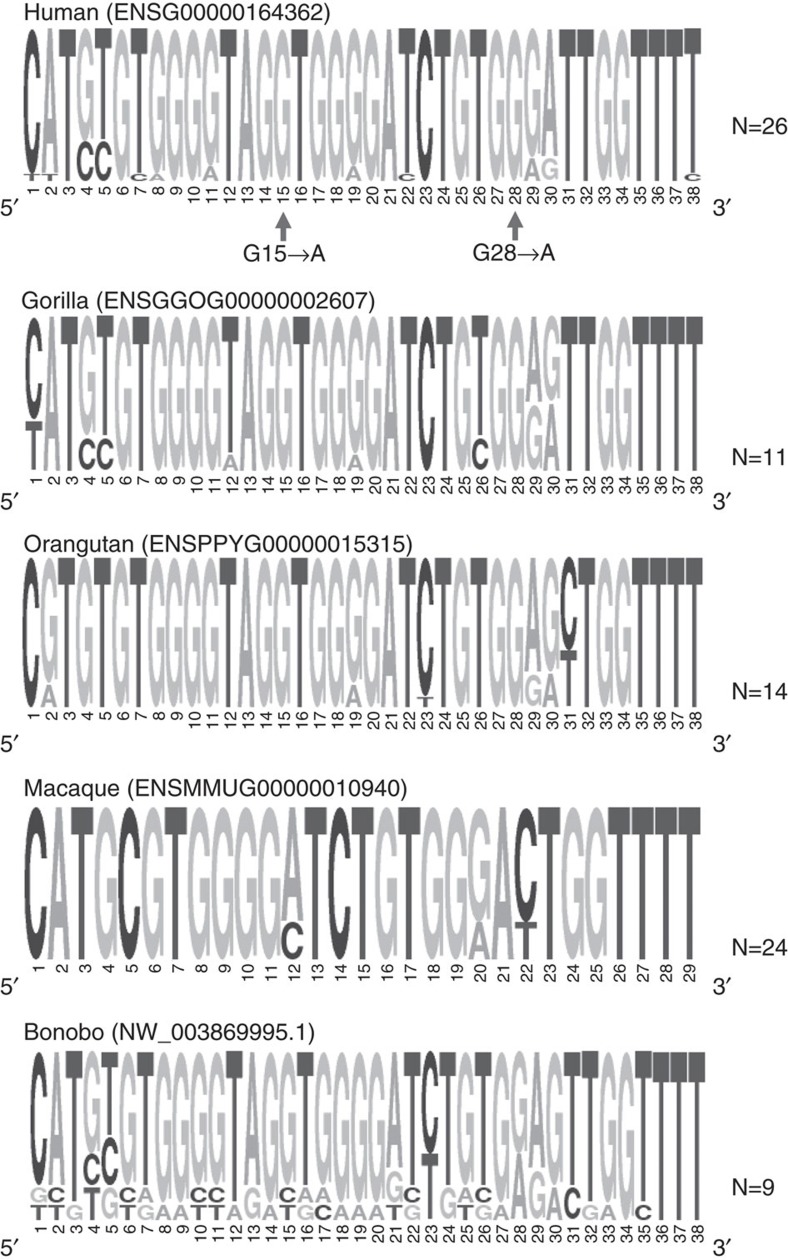
Conservation of the 38 bp consensus repeat. The sequence logo^21^ is shown for old-world primates along with the number of repeats reported for each species. The two single nucleotide mutations introduced in the human sequence, each of which abolished minus beta splicing, are indicated by arrows in the top panel.

**Figure 4:**
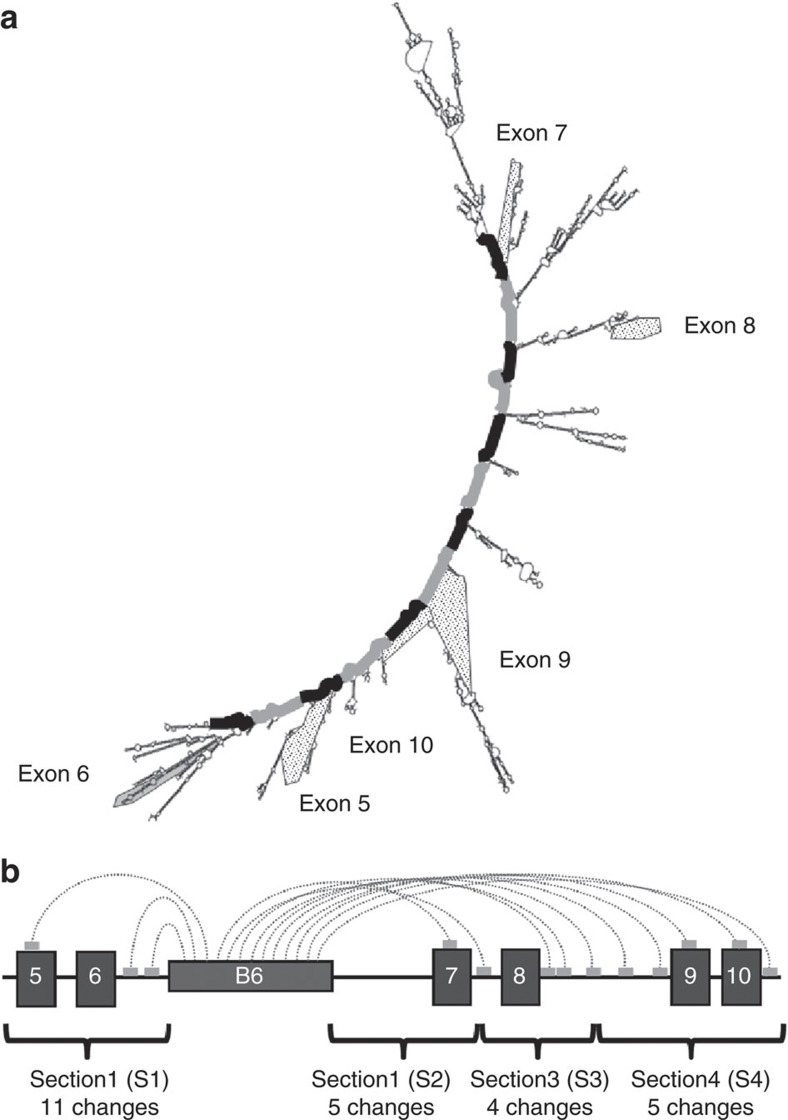
The block 6 repeats are predicted to hybridize along the distal portions of the *TERT* pre-mRNA. (**a**) Mfold analysis predicts that the block 6 repeats (marked by alternating black and grey to represent each 38 nucleotide repeat) hybridize along the distal portions of the *TERT* pre-mRNA to create an elongated structure that puts exon 6 and exon 9 closer together than exons 6 and 7, thereby potentially promoting minus beta splicing. (**b**) Shows a linear representation of the pre-mRNA with the predicted approximate location of each repeat hybridizing along the *TERT* pre-mRNA indicated as light grey boxes. The borders of the sections were determined by useful restriction sites. The number of compensatory mutations introduced within each section is indicated.

The 38 bp repeats in telomerase are a VNTR containing 18–38 repeats with minimal sequence variability in humans^16^. The sequence is conserved but the number of repeats varies greatly in different primate species (bonobo having the least with nine repeats, see Fig. 3). We previously showed that 11 but not 5 repeats could induce exons 7 and 8 skipping, suggesting that a minimal number of repeats is necessary^15^. Mfold analysis suggests that RNA:RNA pairing between the repeats and distal portions of *TERT* pre-mRNA could bring exon 6 and 9 closer together than in a linear configuration (Fig. 4a), which is lost with fewer than nine repeats. Two different single-nucleotide mutations within the repeat sequence (G28→A and G15→A) each abolished the ability of the repeats to induce exon 7 and 8 skipping^15^. This suggests that the tandem repeats in *TERT* intron 6 may rely on RNA:RNA pairing to distal portions of *TERT* pre-mRNA as a mechanism to promote minus beta splicing in human *TERT*.

### Derivation of compensatory mutations

**Figure 5:**
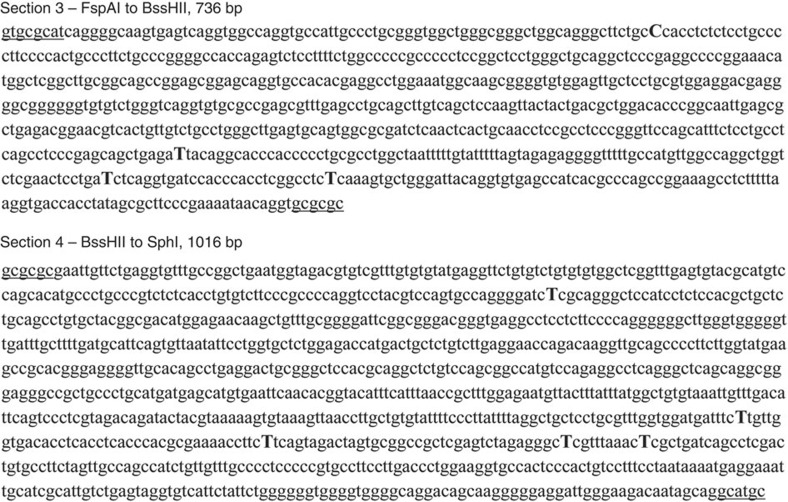
Only combinations of sections 3 and 4 restored pairing interactions. The location of the restriction sites used to substitute different combinations of mutations is underlined at the beginning and end of each sequence. The compensatory mutations introduced into the *hTERT* pre-mRNA are shown as capital letters.

A single mutation in the 38 bp repeat would produce multiple mismatches with many potential targets. This combined with the inability of Mfold to accurately predict large structures posed a challenge to determine which compensatory mutations might restore splicing. Mfold produces numerous secondary folding structures per input sequence based on thermodynamic determinations^17^. Certain predicted secondary structures (in particular, conformations produced by exonic sequences) were relative stable, appearing in all output structures. Other regions were folded into multiple and therefore probably less stable structures. Table 1 summarizes the nucleotides predicted to base pair with the mutated nucleotides of the repeat sequence and the percent of occurrences of such base pairing in multiple Mfold output structures in different segments corresponding to useful restriction sites. Segments containing the predicted complementary mutations were synthesized (Gene Oracle, Mountain View, CA) (Figs 4b and 5) and then used to systematically replace the original sequence in the minigenes containing either mutant or consensus repeats. The high GC content of segment 2 prevented its synthesis so it was not available for replacement studies.

### Compensatory mutations restore function of mutant repeats

**Figure 6:**
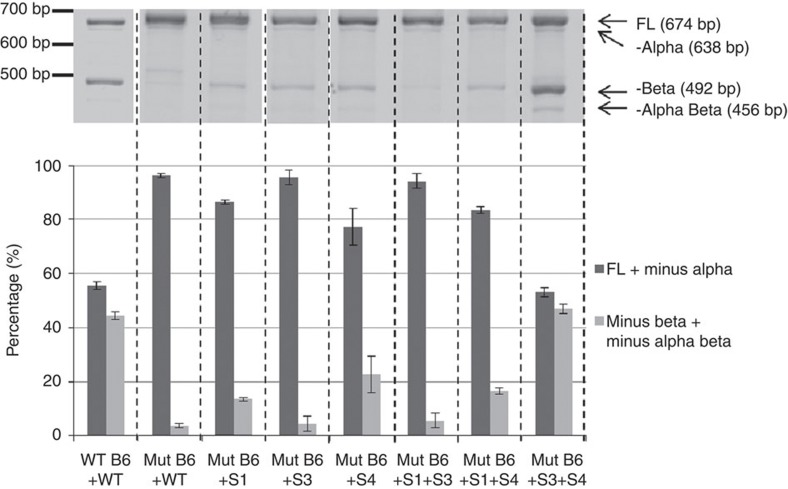
Compensatory mutations within sections 3 and 4 together restore minus beta splicing of the mutant block 6 repeats. The splicing of minigenes containing wild-type or mutant repeats, with or without replacement of sections containing compensatory mutations, are shown together with the quantification of the full-length and minus beta isoforms. Alternative splicing is abolished using a block 6 repeat sequence containing a single-nucleotide mutation, which is restored by introducing compensatory mutations when sections 3 and 4 are combined. Data are from Hela FRT clone 8 cells stably transfected with the minigenes (*n*=3). Error bars represent mean±standard deviation. The results are replicated in three separate transfections in two Hela FRT clones. Minus alpha is a minor alternative splice form that skips the first 36 nt of exon 6. Images of the full-length gels are provided in Supplementary Figs 1 and 2.

**Figure 7:**
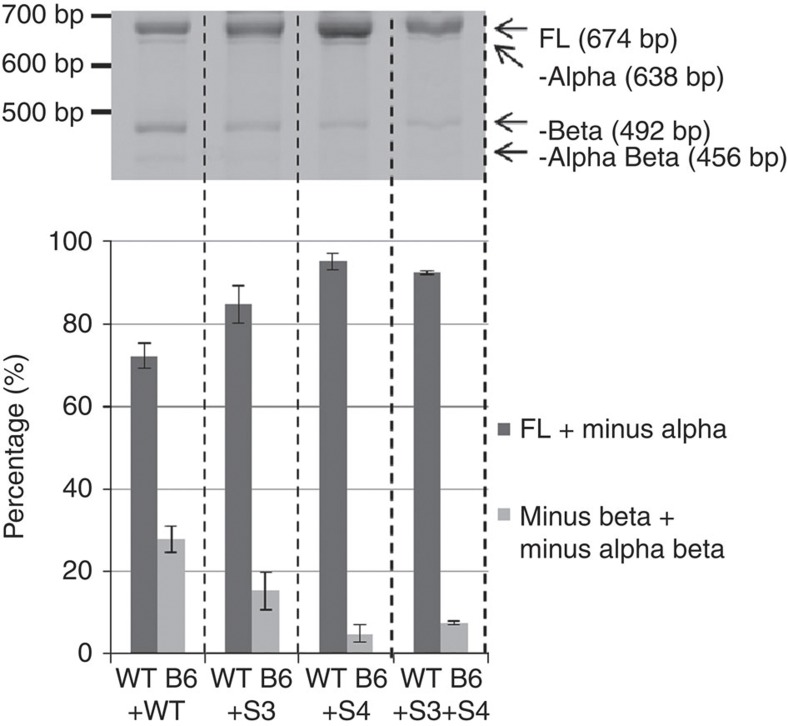
Compensatory mutations within sections 3 and 4 abolish the exon skipping function of the wild-type block 6 repeats. The splicing of minigenes containing wild-type or mutant repeats, with or without replacement of sections containing compensatory mutations, are shown together with the quantification of the full-length and minus beta isoforms. The ability of the wild-type block 6 repeats to promote minus beta splicing is abolished when compensatory mutations in both sections 3 and 4 are introduced to the minigene. Data are from Hela FRT clone 8 cells stably transfected with the minigenes (*n*=3). Error bars represent mean±standard deviation. The results are replicated in three separate transfections in two Hela FRT clones. Images of the full-length gels are provided in Supplementary Figs 1 and 2.

Neither segments 1, 3 or 4 alone compensated the mutant repeats. However, combining segments 3 and 4 resulted in abundant minus beta splicing in repeat mutant G15→A (Fig. 6). Furthermore, the compensatory segments 3 and 4 abolished the ability of wild-type repeats to produce minus beta splicing (Fig. 7). The fact that compensatory mutations restore splicing using mutant repeats and abolish minus beta splicing using wild-type repeats provides the formal proof that RNA:RNA pairing is regulating *TERT* splicing. Segment 4 spans exon 9, thereby potentially influencing splice site availability or exposure of splicing factor binding sites. While segments 3 and 4 together restores minus beta splicing in repeat mutant G15→A, neither segments 1, 3 or 4 alone or in any combination restores minus beta splicing in repeat mutant G28→A. This further suggests that specific RNA:RNA pairings are necessary to promote minus beta splicing.

## Discussion

Prior studies of RNA:RNA pairing have relied on the identification of sequences that are conserved across large evolutionary distances, and usually located close to intron/exon junctions. The lack of conservation of the block 6 repeats, being restricted to old world primates, was unexpected. This is somewhat typical of telomere biology in general, where the constraints appear to be very flexible. While there are many conceptual analogies for their function, the specific solutions to telomere sequence and the proteins that bind and regulate telomere structure vary dramatically between eukaryotes (fission yeast, budding yeast, plants, insects and mammals). For example, although the crystal structures are related, there is virtually no sequence conservation between the *Saccharomyces cerevisiae* Est1–Stn1–Ten1 complex and the mammalian CST–Stn1–Ten1 complex^18^. Telomerase expression correlates with body size where smaller short-lived animals tend to express telomerase ubiquitously while larger long-lived animals repress telomerase expression^19^. The pattern of *TERT* splicing across mammals remains largely unknown. It will be interesting to identify the different splicing patterns and the mechanisms that regulate them in other mammalian species. The involvement of a repetitive element in RNA:RNA pairing is also currently unique to *TERT*. The wide distribution of VNTRs throughout the genome raises the possibility for their involvement with other splicing events.

Altogether, our results suggest that a minimum of nine 38 bp repeats is necessary for RNA:RNA pairing in human telomerase to either change the proximity of exon 6 and 9 splice junctions and/or expose the necessary docking sites for splicing factors or the spliceosome for splice site selection. We have demonstrated the first example of RNA:RNA pairing using a relatively non-conserved repetitive element regulating exon skipping in a mammalian gene. The prevalence of similar regulation in other genes remains to be determined. Manipulating the factors regulating RNA:RNA pairing and the splicing of telomerase may provide additional therapeutic approaches for inhibiting telomerase activity in cancer cells.

## Methods

### Cells

HeLa cervical carcinoma cells were cultured at 37 °C in 5% CO_2_ in 4:1 DMEM:Medium 199, containing 10% calf serum (HyClone, Logan, UT).

### Generation of cells containing FRT site

The Flp-In System (Invitrogen, Carlsbad, CA, USA) was used to generate HeLa cells (purchased from American Type Cell Culture (ATCC)) with a stably integrated FRT site. The population of transfected cells were subcloned and individual clones were screened for a single FRT insertion site by southern blotting. Expression level of the FRT site was determined by beta galactosidase staining. Three Hela clones (no. 1, no. 8, no. 12) were used to confirm the results observed. Data for Hela clone no. 8 is shown in Figs 3 and 4.

### Replacement of sections containing compensatory mutations

**Figure 8:**
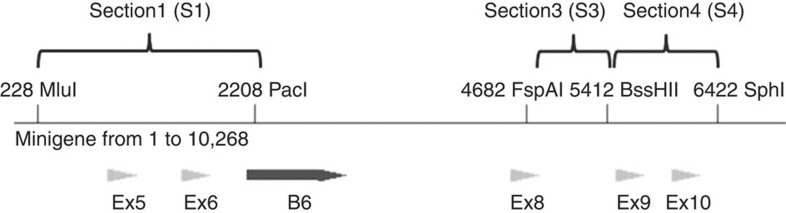
Restriction sites used to introduce compensatory mutations. The nucleotides that appeared most consistently in the Mfold analysis shown in the table were changed to compensatory mutations in blocks of ∼1 kb. Section 2 was not successfully synthesized and was not used in these experiments. Combinations of sections S1, S3 and S4 were tested for their ability to restore splicing using the mutant blocks of repeats. Only sections S3 and S4 successfully restored minus beta splicing.

Three sections containing compensatory mutations were synthesized by Gene Oracle (Mountain View, CA, USA). A *TERT* minigene was previously constructed by inserting portions of the human telomerase sequences into the pcDNA5/FRT expression vector (Invitrogen, Carlsbad, CA, USA). The sequence of the minigene is available upon request. Pre-existing unique restriction sites in the minigene were used to replace the wild-type sections with the sections containing compensatory mutations to avoid additional sequence changes. Section 1 was flanked by a 5′ MluI site and a 3′ PacI site. Section 3 was flanked by a 5′ FspAI site and a 3′ BssHII site. Section 4 was flanked by a 5′ BssHII site and a 3′ SphI site (Fig. 8).

### RT–PCR and splicing analysis

Using the RNeasy Plus Mini Kit (Qiagen, Valencia, CA, USA), RNA was extracted from Hela cells stably expressing the minigene. Complementary DNA was made using Biorad iScript. A minigene specific forward primer 5′-CTGGCTAACTAGAGAACCCACTGC-3′ and a cy5-labelled reverse primer 5′- AGGCTGCAGAGCAGCGTGGAGAGG-3′ were used to examine the splicing of the *hTERT* minigene using Taq polymerase PCR (Promega, Madison, WI). The reaction was initially denatured at 94 °C for 3 min, then denatured at 94 °C for 30 s, annealed at 61 °C for 30 s and extended at 72 °C for 1 min for 30 cycles, with a final extension at 72 °C for 10 min. The PCR product was resolved on a 5% denaturing polyacrylamide gel and visualized at 650 nm.

## Author contributions

M.S.W., J.W.S. and W.E.W. designed the study. M.S.W. performed the experiments and prepared the manuscript. J.W.S. and W.E.W. supervised the data analysis and edited the manuscript.

## Additional information

**How to cite this article:** Wong, M. S. *et al.* Regulation of human telomerase splicing by RNA:RNA pairing. *Nat. Commun.* 5:3306 doi: 10.1038/ncomms4306 (2014).

## Supplementary Material

Supplementary Figures 1-2

## Figures and Tables

**Table 1:** High frequency conserved interactions predicted by Mfold.

**Section 1** **ex5-in5-ex6-in6**		**Section 2** **in6-ex7-in7**		**Section 3** **in7-ex8-in8**		**Section 4** **in8-ex9-in9-ex10**	
**Pairing nucleotide**	**% Output structures**	**Pairing nucleotide**	**% Output structures**	**Pairing nucleotide**	**% Output structures**	**Pairing nucleotide**	**% Output structures**
1332C	24	3839C	69	4498C	100	4768C	100
580C	40	3767C	69	4470C	100	5390C	55
526C	24	3626C	72	4376T	100	5304T	90
799C	7	3054C	97	3943T	28	5343C	21
53T	7	2712C	69			5380C	14
632C	3						
447C	7						
202C	7						
105T	3						
555C	7						
90C	3						
467T	3						

Mfold analysis suggested that the consensus 38 bp block of repeats in *TERT* intron 6 consistently interacts with particular residues of the *TERT* pre-mRNA. Compensatory mutations were designed for the nucleotides showing the highest frequency among the output structures. The exact borders of the sections shown above do not correspond to the borders of the regions synthesized, which were chosen so that convenient restriction sites could be used to introduce the modified pre-mRNA.
