# Supplementary material for: Regulation of human telomerase splicing by RNA:RNA pairing
Source: Nat Commun. 2014 Feb 28;5:3306. doi: 10.1038/ncomms4306 (PMC3948165; doi:10.1038/ncomms4306)
Supplement: Supplementary Figures 1-2 [file ncomms4306-s1.pdf]

Supplementary Figures

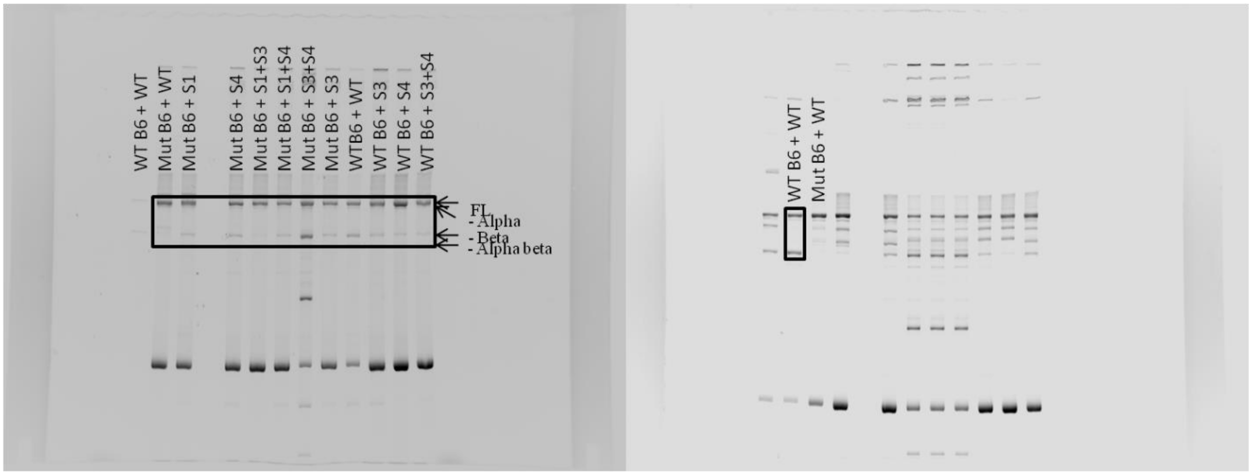

Supplementary Figure 1. Original gel images used in Figure 6 and 7. On the left is the original gel used for Figures 6 and 7. The box shows the region cropped for the figures. Due to the faintness of lane 1 (WT B6 + WT), we also display a different WT B6 + WT on the right for comparison. Lane 9 was loaded out of order and was reordered in Figure 6.

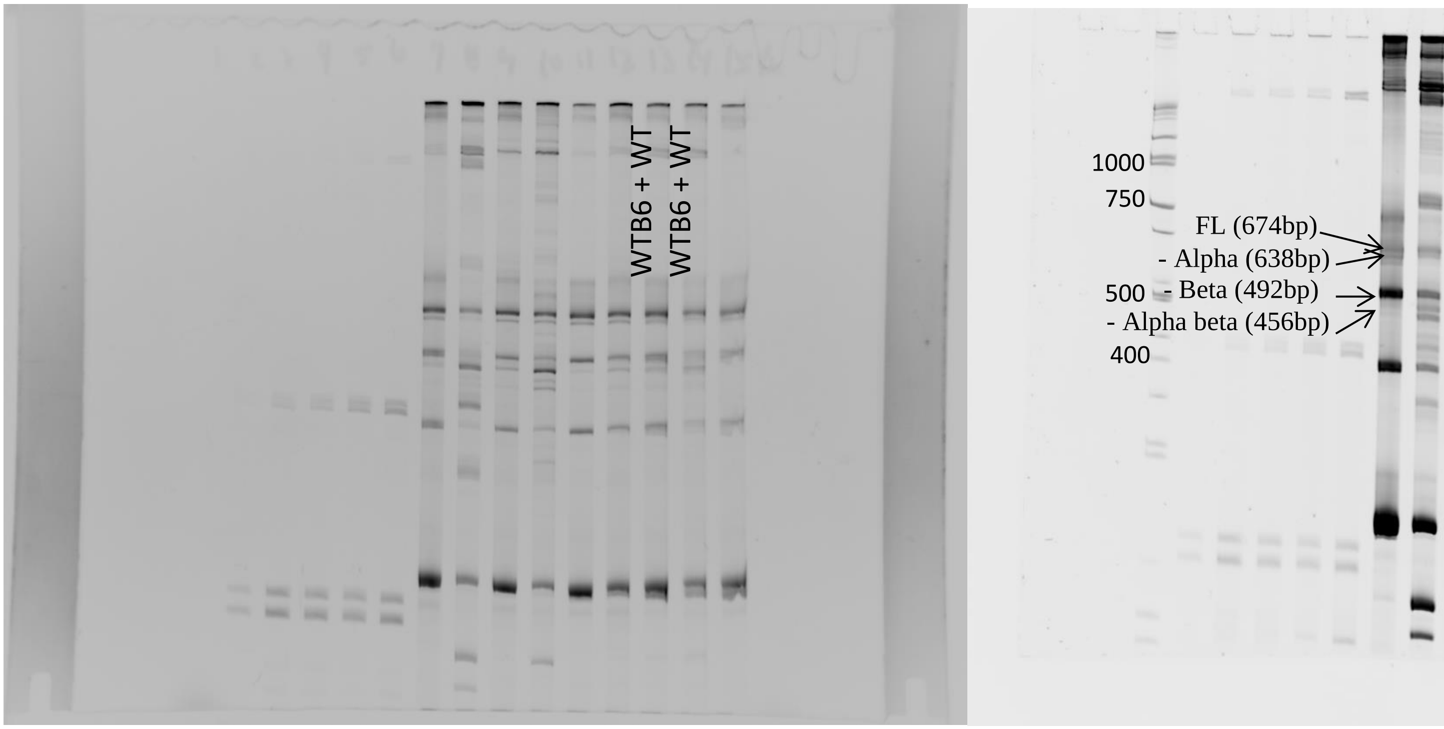

Supplementary Fig. 2. Example of Visualization of the molecular weight marker that is post-stained using GelRed. The minigene in the transfected cells is PCR-amplified using a minigene-specific forward primer (FRT1) and a Cy5-labelled reverse primer within exon 9 and ran on a denaturing 5% polyacrylamide gel. Shown on the left is an experiment using minigenes unrelated to the paper, captured using the Cy5 channel. Subsequently, the gel is stained in GelRed for 1 hour to obtain the image on the right with the ladder. The ladder used is the Hi-Lo ladder (Bionexus Inc). Since all of the minigene samples we analyze produce the same distinct pattern of FL, minus alpha, minus beta, and minus alpha beta splicing pattern, staining of the ladder is usually not performed.
